# Nomogram to predict hemorrhagic transformation for acute ischemic stroke in Western China: a retrospective analysis

**DOI:** 10.1186/s12883-022-02678-2

**Published:** 2022-04-26

**Authors:** Keming Zhang, Jianfang Luan, Changqing Li, Mingli Chen

**Affiliations:** 1 School of Clinical Medicine, Chongqing Medical and Pharmaceutical College, Chongqing, China; 2 Department of Neurology, Chongqing Sanbo Changan Hospital, 65 Jianxin East Road, Jiangbei District, Chongqing, 400023 China; 3 Department of Neurology, The Second Affiliated Hospital of Chongqing Medical University, Chongqing, China

**Keywords:** Hemorrhagic transformation, Acute ischemic stroke, Nomogram, NIHSS, Thrombolysis

## Abstract

**Background and purpose:**

Hemorrhagic transformation (HT) is the most alarming complication of acute ischemic stroke. We aimed to identify risk factors for HT in Chinese patients and attempted to develop a nomogram to predict individual cases.

**Methods:**

A retrospective study was used to collect the demographic and clinical characteristics of ischemic stroke patients at the Second Affiliated Hospital of Chongqing Medical University (development cohort) and Chongqing Sanbo Changan Hospital (validation cohort) from October 2013 to August 2020. Univariate analysis and multivariate analysis were used to identify the risk factors of patients in the development cohort. The nomogram was generated, and internal validation was performed. We used the area under the receiver-operating characteristic curve (AUC-ROC) to assess the discrimination and used the Hosmer–Lemeshow test to calibrate the model. To further verify the predictability and accuracy of the model, we performed an external validation of the patients in the validation cohort.

**Results:**

A total of 570 patients were used to generate the nomogram. After univariate analysis and multivariate logistic regression, the remaining 7 variables (diabetes mellitus, atrial fibrillation, total cholesterol, fibrous protein, cerebral infarction area, NIHSS score and onset-to-treatment) were independent predictors of HT and used to compose the nomogram. The area under the receiver-operating characteristic curve of the model was 0.889 (95% CI, 0.841–0.938), and the calibration was good (*P = 0.487* for the Hosmer–Lemeshow test). The model was validated externally with an AUC-ROC value of 0.832 (95% CI, 0.727–0.938).

**Conclusions:**

The nomogram prediction model in this study has good predictive ability, accuracy and discrimination, which can improve the diagnostic efficiency of HT in patients with acute ischemic stroke.

**Supplementary Information:**

The online version contains supplementary material available at 10.1186/s12883-022-02678-2.

## Introduction

HT refers to intracranial hemorrhage that occurs after acute ischemic stroke as a result of injured blood vessels and restoration of blood perfusion. HT is the most feared complication for acute ischemic stroke. Worldwide, the incidence of HT ranges from 0.6 to 85%, with rates as high as 10 to 48% after thrombolytic therapy [1, 2]. Previous studies have shown that Asian stroke patients may have a higher risk of intracranial hemorrhage than Western populations [3–5]. Symptomatic intracranial hemorrhage (SICH) is the most severe type of HT and is closely related to a poor patient prognosis [6]. A recent study noted that the incidence of SICH in China is 7.3%, which is much higher than the global incidence [7]. By using a continuous score, a nomogram is a statistical instrument that accounts for numerous variables to calculate the probability of a particular outcome for an individual patient [8, 9]. Nomograms are an important part of medical decision-making and have been widely used in cancer and surgery [9, 10]. In recent years, nomograms have been gradually applied to the treatment decision of stroke [11–13]. A recent study confirmed that the nomogram is more reliable than SITS-SICH and SEDAN scores for estimating the risk of SICH [8].

The aim of this study was to identify the risk factors for HT and to develop and validate a nomogram to predict the risk of HT in individual acute ischemic stroke patients.

## Methods

### Study design, participants, and procedures

We performed a retrospective analysis of patients with acute ischemic stroke who were enrolled at the Second Affiliated Hospital of Chongqing Medical University (development cohort) or Chongqing Sanbo Changan Hospital (validation cohort) between October 2013 and March 2020. Informed consent was obtained from all participating patients or their legally authorized representatives.

This study was approved by the institutional review board of each participating institution and in accordance with the tenants of the Declaration of Helsinki.

### Inclusion criteria

We included all the patients with complete data on all the variables included in the nomogram, and those with clinical and radiological data to determine HT. In addition, patients who were age ≥ 18 years, admitted to the hospital no more than 7 days after onset, and met the diagnostic criteria for acute ischemic stroke were included.

### Exclusion criteria

Patients who were age < 18 years, with incomplete data, history of hematological diseases with bleeding tendency and accompanied by severe physical diseases were excluded from the present study.

### Definitions

Diagnostic criteria for acute ischemic stroke were as follows:(1) acute onset; (2) focal neurological deficit (weakness or numbness of one side of the face or limbs, language disorders, etc.), and a few are full-scale neurological deficits; (3) lesions present on imaging or symptoms and signs lasting for longer than 24 h; (4)exclusion of nonvascular etiology; and (5) exclusion of intracerebral hemorrhage by imaging [14].

Diagnostic criteria for HT: (1) no intracranial hemorrhage was found in the first cranial imaging examination after cerebral infarction, and intracranial hemorrhage was found in the second cranial imaging examination (the second cranial imaging examination time: normally in one week or immediately in case of clinical worsening); (2) could be determined based on the first cranial imaging examination [15–17]. The imaging results were evaluated by neuroradiologists blinded to patient details, including the clinical characteristics of the stroke.

Classification of HTs: HTs were classified according to the European Cooperative Acute Stroke Study II (ECASS II). (1) HI1, small petechiae along the margins of the infarct; (2) HI2, confluent petechiae within the infarcted area but no space occupying effect; (3) PH1, blood clots in ≤30% of the infarcted area with some slight space-occupying effect; (4) PH2, blood clots in > 30% of the infarcted area with a substantial space-occupying effect [18, 19]. These results were determined jointly by radiologists and neurologists.

Diagnostic criteria for SICH: any type of intracerebral hemorrhage with an increase in the National Institutes for Health Stroke Scale (NIHSS) score of ≥4 points from baseline [8, 18, 19].

NIHSS score: after admission, the NIHSS score was determined before treatment.

THRIVE score: after admission, the THRIVE score was performed before treatment. 1 point for history of hypertension, 1 point for history of diabetes, 1 point for history of atrial fibrillation, 1 point for age 60–79 years old, 2 points for age ≥ 80 years old, 2 points for NIHSS score 11–20, 4 points for NIHSS score ≥ 21.

Onset-to-treatment (OTT): the time from the onset of the patient’s symptoms to admission to the hospital for treatment. Treatment includes the endovascular treatment and prescribed medications (e.g., antiplatelet, oral anticoagulant, etc.).

Thrombolysis or ET: thrombolysis--the patient received intravenous thrombolysis with rt-PA or urokinase; ET-- endovascular treatment.

### Data collection and quality control

Risk factors related to HT were determined based on evidence-based medical literature retrieval results and clinical practice. Patient medical records were gathered retrospectively by two neurologists. All researchers had relevant professional knowledge and began data collection after completing unified training. When screening the research subjects, the inclusion and exclusion criteria were strictly followed, and preliminary screening was conducted by double review. After screening, the eligible cases with complete data were double checked and entered to ensure the completeness and accuracy of the data. The following data were collected: demographic data, medical history, baseline data, imaging data, THRIVE score and NIHSS score, and treatment data.

### Statistical analysis

The patient data in the development cohort were used to develop the prediction model, and the patient data in the validation cohort were used to validate the model.

The data are presented as the median (interquartile range), the means ± standard deviation (SD) or number (%).Mann–Whitney tests were used for continuous variables, and Fisher’s exact tests or the χ2 tests were used for categorical variables. SPSS 23.0 was used for statistical analyses.

All variables with a probability value < 0.10 in the univariate analysis or that have been consistently identified as affecting outcome (based on our clinical experience or the published work) entered into a multivariate logistic regression analysis using a forwards stepwise method. The collinearity of combinations of variables in the development cohort was evaluated by the variation inflation factors (< 2 being considered not significant) and condition index (< 30 being considered not significant). We used the AUC-ROC to assess the discrimination of the prediction model and used the Hosmer-Lemeshow test to calibrate the model.

Then, we performed external validation to assess the accuracy of the prediction model obtained from the development cohort in the validation cohort by the AUC-ROC and calibration.

R software (version 4.0.3) was used to build the nomogram prediction model. The nomogram converts each independent risk factor included in the model into an assessment point system. The total points obtained determine the final risk assessment value.

## Results

A total of 392 patients from the Second Affiliated Hospital of Chongqing Medical University were included in the development cohort, and 178 patients from Chongqing Sanbo Changan Hospital were included in the validation cohort. The demographics and clinical characteristics of the patients in the two cohorts are shown in Table 1. A total of 62(10.9%) patients had HT after acute ischemic stroke. Forty-eight (12.2%) patients were in the development cohort, and 14 (7.9%) were in the validation cohort (Supplementary Table 1).

**Table 1:** Demographics and Clinical Characteristics of the patients in the development and validation cohorts

	development cohort (*n* = 392)	validation cohort (*n* = 178)
Demographics		
Age, y, median (IQR)	70(60–79)	72(61–81)
Age > 60 y, n (%)	287(73.2)	136(76.4)
Male sex, n (%)	236(60.2)	102(57.3)
Medical history, n (%)		
Hypertension	288(73.5)	131(73.6)
Diabetes mellitus	125(31.9)	62(34.8)
Previous stroke	53(13.5)	50(28.1)
Atrial fibrillation	80(20.9)	36(20.2)
Antiplatelet	53(13.5)	48(27.0)
Oral anticoagulant	13(3.3)	21(11.8)
Smoking current	147(37.5)	62(34.8)
Baseline data, median (IQR)		
Systolic BP, mm Hg,	153(137–171)	157(138–175)
Diastolic BP, mm Hg,	88(80–99)	88(77–99)
Temperature,°C	36.5(36.4–36.7)	36.5(36.2–36.7)
Glucose, mmol/L	6.32(5.15–8.31)	7.47(6.15–9.59)
Total cholesterol (TC), mmol/L	4.78(4.06–5.45)	4.81(4.08–5.58)
Triglyceride (TG), mmol/L	1.33(1.01–1.88)	1.51(1.06–2.33)
High-density lipoprotein (HDL), mmol/L	1.06(0.91–1.25)	1.39(1.14–1.65)
Low-density lipoprotein (LDL), mmol/L	2.90(2.20–3.51)	2.76(2.17–3.31)
Blood platelet (PLT), × 10^9^/L	179.00(135.25–215.75)	194.00(154.75–226.50)
Prothrombin time (PT)%	99.00(90.00–109.00)	91.64(81.34–101.97)
APTT, s	35.20(32.40–37.88)	28.40(26.00–30.53)
INR	1.00(0.95–1.07)	1.04(0.99–1.10)
Fibrous Protein (Fib), g/L	3.29(2.84–3.94)	3.04(2.57–3.52)
Imaging data, median (IQR)		
Cerebral Infarction Area (CIA), cm^2^	3.00(1.00–9.69)	3.16(0.99–19.09)
Cerebral Infarction Volume (CIV), cm^3^	3.87(0.78–19.31)	3.78(0.72–43.28)
Scores		
THRIVE score, median (IQR)	2.00(2.00–3.75)	3.00(2.00–4.00)
NIHSS score, median (IQR)	5(3–9)	6 (4–16)
NIHSS score > 20, n (%)	11(2.8)	22(12.7)
Treament		
onset-to-treatment (OTT), h, median (IQR)	13.50(5.00–48.00)	7.00(2.00–24.00)
Thrombolysis or ET, n (%)	21(5.4)	32(18.5)

*APTT* activated partial thromboplastin time, *INR* international normalized ratio, *NIHSS* National Institutes for Health Stroke Scale

Univariate analysis was performed for 30 variables in the development cohort. *P < 0.1* was considered statistically significant. Fifteen variables (atrial fibrillation *p = 0.000*, systolic BP *p = 0.024*, diastolic BP *p = 0.053*, temperature *p = 0.045*, glucose *p = 0.015*, total cholesterol (TC) *p = 0.004*, prothrombin time (PT)% *p = 0.002*, international normalized ratio (INR) *p = 0.009*, fibrous protein (Fib) *p = 0.003*, cerebral infarction area (CIA) *p = 0.000*, cerebral infarction volume (CIV) *p = 0.000*, THRIVE score *p = 0.000*, NIHSS score *p = 0.000*, NIHSS score > 20 *p = 0.000*, thrombolysis *p = 0.019*) were statistically significant (Table 2). Based on our clinical experience and the published work, we believe that there are 8 additional variables (hypertension, diabetes mellitus, previous stroke, antiplatelet, oral anticoagulant, blood platelet (PLT), activated partial thromboplastin time (APTT), OTT) that may affect the outcome. Therefore, a total of 23 variables were entered into the multivariate logistic regression analysis. Only 8 variables remained independent predictors of HT (Supplementary Table 2). However, a significant statistical collinearity was observed for 2 variables (cerebral infarction area VIF = 11.283, cerebral infarction volume VIF = 11.071) (Supplementary Tables 3, 4). Then, we performed a Pearson correlation analysis for the two variables. There was a significant correlation between the two variables (*p = 0.952*). According to our clinical experience, cerebral infarction volume and HT should not be negatively correlated. Therefore, we eliminated the variable of cerebral infarction volume and replaced it with cerebral infarction area. Multivariate regression analysis was conducted again, and the results are shown in Table 3. Finally, seven independent predictors (diabetes mellitus [OR, 2.483; 95% CI, 1.128–5.468; *P = 0.024*], atrial fibrillation [OR, 6.645; 95% CI, 2.948–14.976; *P = 0.000*], TC [OR, 0.669; 95% CI, 0.474–0.943; *P = 0.022*], Fib [OR, 1.498; 95 CI, 1.083–2.072; *P = 0.015*], cerebral infarction area [OR, 1.064; 95% CI, 1.028–1.100; *P = 0.000*], NIHSS score [OR, 1.106; 95% CI, 1.033–1.184; *P = 0.004*], and OTT [OR, 1.024; 95% CI, 1.010–1.039; *P = 0.001*]) were used to build the nomogram model.

**Table 2:** Univariate analysis of the variables associated with HT in the development cohort

	HT (*n* = 48)	Non-HT (*n* = 344)	*P* Value
Demographics			
Age, y, median (IQR)	71.50(62.00–77.00)	70.00(59.25–79.00)	0.672
Age > 60 y, n (%)	39(81.3)	248(72.1)	0.180
Male sex, n (%)	29(60.4)	207(60.2)	0.974
Medical history, n (%)			
Hypertension	32(66.7)	256(74.4)	0.254
Diabetes mellitus	18(37.5)	107(31.1)	0.373
Previous stroke	7(14.6)	46(13.4)	0.818
Atrial fibrillation	28(58.3)	52(15.1)	0.000*
Antiplatelet	4(8.3)	49(14.2)	0.262
Oral anticoagulant	3(6.3)	10(2.9)	0.435
Smoking current	14(29.2)	133(38.7)	0.203
Baseline data, median (IQR)			
Systolic BP, mm Hg,	140.00(130.00–167.00)	154.50(139.25–172.00)	0.024*
Diastolic BP, mm Hg,	81.00(75.25–92.00)	80.00(80.00–100.00)	0.053*
Temperature,°C	36.55(36.40–36.90)	36.50(36.40–36.68)	0.045*
Glucose, mmol/L	6.61(5.93–11.43)	6.23(5.07–7.91)	0.015*
TC, mmol/L	4.22(3.81–5.09)	4.83(4.11–5.47)	0.004*
TG, mmol/L	1.28(0.94–1.69)	1.36(1.01–1.90)	0.212
HDL, mmol/L	1.04(0.85–1.27)	1.06(0.91–1.25)	0.411
LDL, mmol/L	2.72 ± 0.90	2.91 ± 0.95	0.200
PLT, ×10^9^/L	158.50(127.50–211.25)	180.00(141.00–216.00)	0.169
PT%	93.50(84.25–104.50)	100.50(92.00–110.00)	0.002*
APTT, s	35.60(32.43–38.45)	35.20(32.40–37.80)	0.359
INR	1.04(0.97–1.11)	1.00(0.95–1.06)	0.009*
Fib, g/L	3.59(3.08–4.66)	3.27(2.82–3.87)	0.003*
Imaging data, median (IQR)			
Cerebral Infarction Area (CIA), cm^2^	16.43(8.81–29.52)	2.22(0.92–7.10)	0.000*
Cerebral Infarction Volume (CIV), cm^3^	32.58(15.4179.20)	2.51(0.62–12.81)	0.000*
Scores			
THRIVE score, median (IQR)	4.00(3.00–5.75)	2.00(2.00–3.00)	0.000*
NIHSS score, median (IQR)	11.50(6.25–15.00)	4.00(3.00–8.00)	0.000*
NIHSS score > 20, n (%)	7(14.6)	4(1.2)	0.000*
Treament			
OTT, h, median (IQR)	15.00(3.00–48.00)	13.50(5.00–48.00)	0.757
Thrombolysis or ET, n (%)	6(12.5)	15(4.4)	0.019*

*TC* total cholesterol, *TG* triglyceride, *HDL* high-density lipoprotein, *LDL* low-density lipoprotein, *PLT* blood platelet, *PT* Prothrombin time, *APTT* activated partial thromboplastin time, *INR* international normalized ratio, *Fib* fibrous protein, *NIHSS* National Institutes for Health Stroke Scale, *OTT* onset-to-treatment, * *p<0.1*

**Table 3:** Multivariable logistic regression analysis of the variables associated with HT in the development cohort

	Regression coefficient	Standard Error	Odds Ratio	95% Confidence Interval	*P* value
Diabetes mellitus	0.910	0.403	2.483	(1.128–5.468)	0.024
Atrial fibrillation	1.894	0.415	6.645	(2.948–14.976)	0.000
TC	−0.402	0.175	0.669	(0.474–0.943)	0.022
Fib	0.404	0.166	1.498	(1.083–2.072)	0.015
Cerebral infarction Area	0.062	0.017	1.064	(1.028–1.100)	0.000
NIHSS score	0.101	0.035	1.106	(1.033–1.184)	0.004
onset-to-treatment (OTT)	0.024	0.007	1.024	(1.010–1.039)	0.001

*TC* total cholesterol, *Fib* fibrous protein, *NIHSS* National Institutes for Health Stroke Scale

**Fig. 1:**
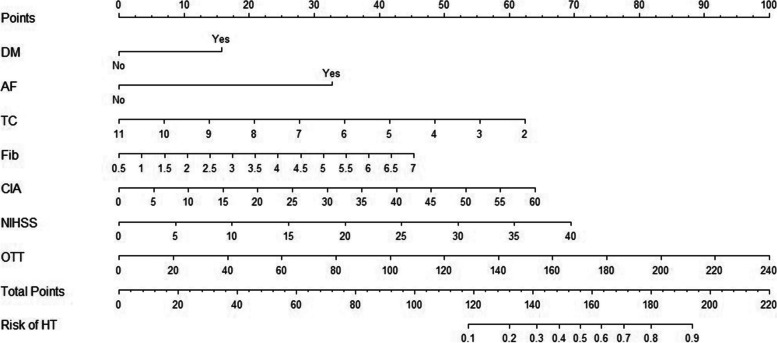
The nomogram prediction model. To use, mark an individual’s DM, draw a vertical line up to the points axis to establish the score associated with DM. Repeat this process for the other six predictors. Add the scores for each predictor together and mark the total score on the total points axis. A vertical line is drawn down to the risk of the HT axis to obtain the probability. DM = diabetes mellitus. AF = atrial fibrillation. TC = total cholesterol. Fib = fibrous protein. CIA = cerebral infarction area. NIHSS = National Institutes of Health Stroke Scale score. OTT = onset-to-treatment

Each of the seven independent predictors was converted into a graphic preliminary score from 0 to 100, which was then summed to obtain a total score. Finally, the total score was converted into an individual risk of HT after acute ischemic stroke (from 0 to 100%) (Fig. 1).

**Fig. 2:**
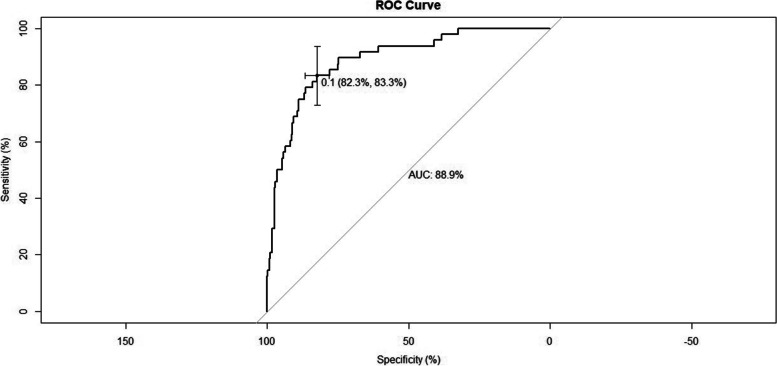
The discriminative performance of the nomogram prediction model. The AUC-ROC of the model was 0.889 (95% CI, 0.841–0.938), and when *P = 0.1*, the specificity and sensitivity were 82.3 and 83.3% respectively

**Fig. 3:**
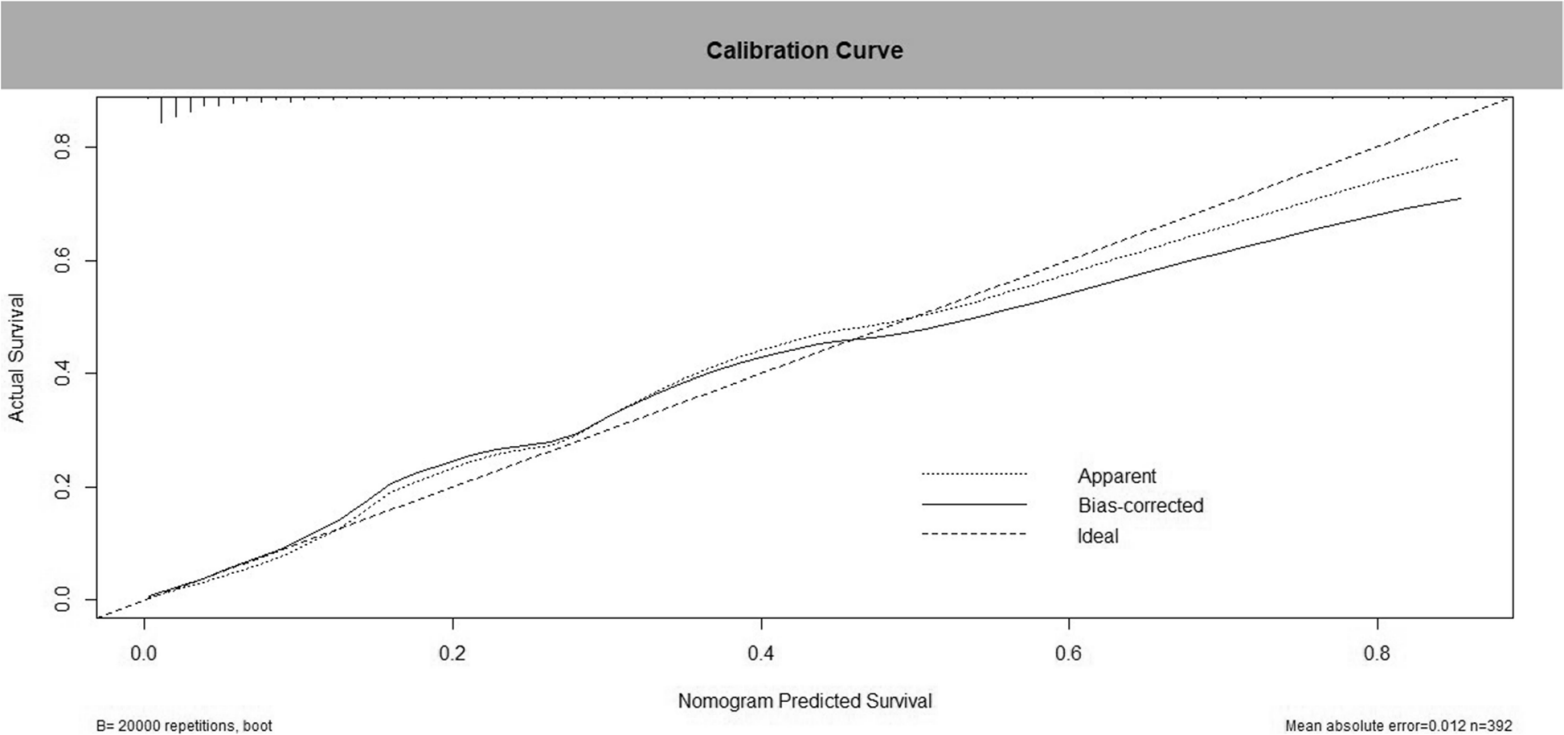
Calibration curve for internal (development cohort) validation of the nomogram prediction model. The ideal line indicates the reference line, indicating where an ideal nomogram would lie and the apparent line indicates the actual nomogram prediction line. The calibration was good (7.466; *P = 0.487* for the Hosmer–Lemeshow test)

The nomogram model was internally validated using 20,000 bootstrap replicates. The AUC-ROC of the model was 0.889 (95% CI, 0.841–0.938), and the calibration was good (7.466; *P = 0.487* for the Hosmer–Lemeshow test) (Figs. 2 and 3). In the validation cohort, the nomogram model was externally validated. Compared with the discriminative performance in the development cohort, the AUC-ROC value was similar (0.831; 95% CI, 0.724–0.938) (Supplementary Fig. 1). Therefore, the nomogram prediction model in this study has good predictive ability, good accuracy and good discrimination, which can improve the diagnostic efficiency of HT in patients with acute ischemic stroke.

## Discussion

Many papers have been published on HT after ischemic stroke and many predictors of HT have been identified. Most of these studies focus on specific populations of patients with symptomatic intracranial hemorrhage that occurs after intravenous thrombolysis in ischemic stroke patients. These studies provide individualized risk prediction for HT after intravenous thrombolysis in ischemic stroke patients, which is conducive to the choice of clinical treatment and prognosis.

HT is the most alarming complication for acute ischemic stroke. Previous studies reported that the incidence rate of HT was 0.6–85% [1, 2]. In different studies, the significant differences in the incidence of HT may be related to sample selection bias, different assessment methods, inconsistent diagnostic criteria, different times of the initial diagnosis, and bias of researchers’ subjectivity. Among the 392 patients with acute ischemic stroke in this study, the incidence of hemorrhagic conversion was 11%. Among them, the incidence of hemorrhagic transformation in patients in the Second Affiliated Hospital of Chongqing Medical University was 12.2%, and that in Chongqing Sanbo Changan Hospital was 8%. The incidence of HT was similar in the two hospitals.

According to previous studies, our clinical work experience and the characteristics of the Chinese population, 30 HT-related risk factors were selected for inclusion in this study. After univariate analysis and the initial multivariate regression analysis, only the remaining 8 risk factors were independent predictors of HT (shown in Supplementary Table 2). We found that among the 8 risk factors, CIV and TC were negatively correlated with the incidence of HT; that is, CIV and TC were protective factors. However, based on our clinical experience and relevant literature reports [20, 21], CIV and CIA should be positively correlated with the incidence of HT. After repeated verification, we found that the CIV and CIA were collinear and highly correlated (*p = 0.952*). Therefore, only the CIA, which is more realistic, was retained, and the CIV was excluded.

TC is a decisive factor in maintaining the integrity of cell fluidity, and it is involved in the composition of cell membrane receptors, transport and immune function. An appropriate TC level is of significant for maintaining the integrity of the small vessel wall [22]. In addition, a study showed that low levels of TC may increase the risk of HT. [23] A recent study suggested that TC level was an independent prognostic factor for intravenous thrombolysis outcome [24]. These studies indicate that TC is a protective factor for HT, which is consistent with the results of this study.

Among the eight risk factors, atrial fibrillation was the most associated with HT incidence, which may be related to the increasing incidence of atrial fibrillation and cardiogenic cerebral embolism in China [25]. Several studies found that acute ischemic stroke patients with atrial fibrillation have a higher risk of HT than patients without atrial fibrillation [20, 26, 27]. Recent studies [28, 29] have suggested that atrial fibrillation was an independent risk factor for HT after intravenous thrombolysis in stroke patients and may be more important in China than in Western populations [3]. In general, the most common etiology of acute ischemic stroke patients with atrial fibrillation is cardiogenic thrombus [28]. These patients have a high degree of vascular occlusion, large cerebral infarction area, poor ability to reconstruct collateral circulation, and severe damage to the blood–brain barrier. The exudation of inflammatory transmitters, matrix metalloproteinases (MMPs) and free radicals increases the damage to blood vessels, which makes it easier to cause bleeding when restoring blood perfusion [30–32]. One study showed that 65% of patients with acute cardioembolic stroke had spontaneous bleeding in the infarcted lesions within 48 h after onset, which may be related to the spontaneous dissolution caused by poor stability of cardiogenic thrombus [33]. This finding was consistent with previous studies [34, 35].

NIHSS scores have been widely used to assess stroke severity, and higher scores indicate more severe neurological deficits resulting from stroke. In general, patients with higher NIHSS scores tend to have poor pial collateral formation, which is associated with larger infarct volumes [36–38]. One study showed that the NIHSS scores reflected the initial cerebral lesion volume and the functional impact of this lesion, and the rate of functional independence declined with increasing initial NIHSS scores [39]. Many previous studies concluded that NIHSS score at admission is an independent risk factor for predicting HT and has good performance [7, 37, 40]. The results of the present study showed that NIHSS score at admission was significantly correlated with the incidence of HT, and was an independent risk factor for HT, which was consistent with previous literature reports. In addition, another study demonstrated that only 3% of patients developed HT when the NIHSS score was less than 10 [41].

When acute ischemic stroke is accompanied by hyperglycemia, glycolysis can produce cytotoxic acidic products, which can aggravate the swelling of vascular endothelial cells, reduce microcirculation, aggravate vascular damage, and further exacerbate the symptoms of neurological deficit [42]. A recent study showed that hyperglycemia at admission is significantly associated with unfavourable outcomes for patients with acute ischemic stroke but does not increase the risk of symptomatic intracranial hemorrhage [43]. In the present study, univariate analysis showed that hyperglycemia was associated with HT, but multivariate regression analysis showed that hyperglycemia was not an independent risk factor for HT, which was consistent with the literature reports. In addition, although a history of diabetes was not a risk factor for HT in the univariate analysis in our study, we also entered the history of diabetes into the multivariate regression analysis based on our clinical experience, and the results showed that diabetes mellitus was an independent risk factor for HT. This result suggests that special attention should be given to the possibility of HT in patients with diabetes mellitus, whether they have hyperglycemia at admission.

Previous studies showed that high Fib levels were associated with a higher incidence of HT in acute ischemic stroke patients [44, 45]. A recent study demonstrated that high Fib levels were independently associated with HT and were significantly associated with unfavorable long-term outcomes in nonatrial fibrillation patients [46]. In our study, we found that higher Fib may be an independent risk factor for HT among acute ischemic stroke patients, which is congruent with these reports. However, another study found that low Fib levels were a risk factor for HT. [47] Therefore, the specific relationship between Fib and HT is still unclear and needs to be further explored.

Our study has several strengths. First, the study is not a single-center study; it included two general hospitals at different levels. Therefore, the sample of patients included in the study is large and representative. Second, the nomogram in this study was not derived from a large development cohort but was also externally validated in a validation cohort. The acceptable performance of the nomogram in the validation cohort lends credibility to its usefulness in different hospitals. Third, all the variables included in the nomogram are easily ascertainable clinical characteristics. It is very convenient for clinicians to conduct rapid evaluation. These features will make our nomogram a useful clinical instrument.

Our study has some limitations. First, it is based on a retrospective analysis, thus having the limitations of such a study design. Second, the subjects of this study are mainly Chinese, which may need to be validated in more different populations. However, the incidence of acute ischemic stroke in China is much higher than that in the world, and the incidence of HT is also high in China. Therefore, the nomogram based on our data should be useful in the clinical setting. Although 30 variables were comprehensively collected for the study, some risk factors associated with HT were not included due to the lack of data. However, more important and easily available factors have been included, which is also conducive to clinical applications.

In conclusion, our nomogram was developed and externally validated in different hospitals to predict the risk of HT after acute ischemic stroke. Diabetes mellitus, TC, Fib, CIA, NIHSS score and OTT were independent predictors of HT. Our nomogram can be applied, easily and quickly, in the clinical setting.

## Supplementary Information

**Additional file 1.**

**Additional file 2.**

**Additional file 3.**

**Additional file 4.**

**Additional file 5.**

## Data Availability

Further clinical data are available from the corresponding author upon reasonable request.
