# Supplementary material for: Nomogram to predict hemorrhagic transformation for acute ischemic stroke in Western China: a retrospective analysis
Source: BMC Neurol. 2022 Apr 26;22:156. doi: 10.1186/s12883-022-02678-2 (PMC9040382; doi:10.1186/s12883-022-02678-2)
Supplement: Supplementary file 1 — Additional file 1. [file 12883_2022_2678_MOESM1_ESM.docx]

**
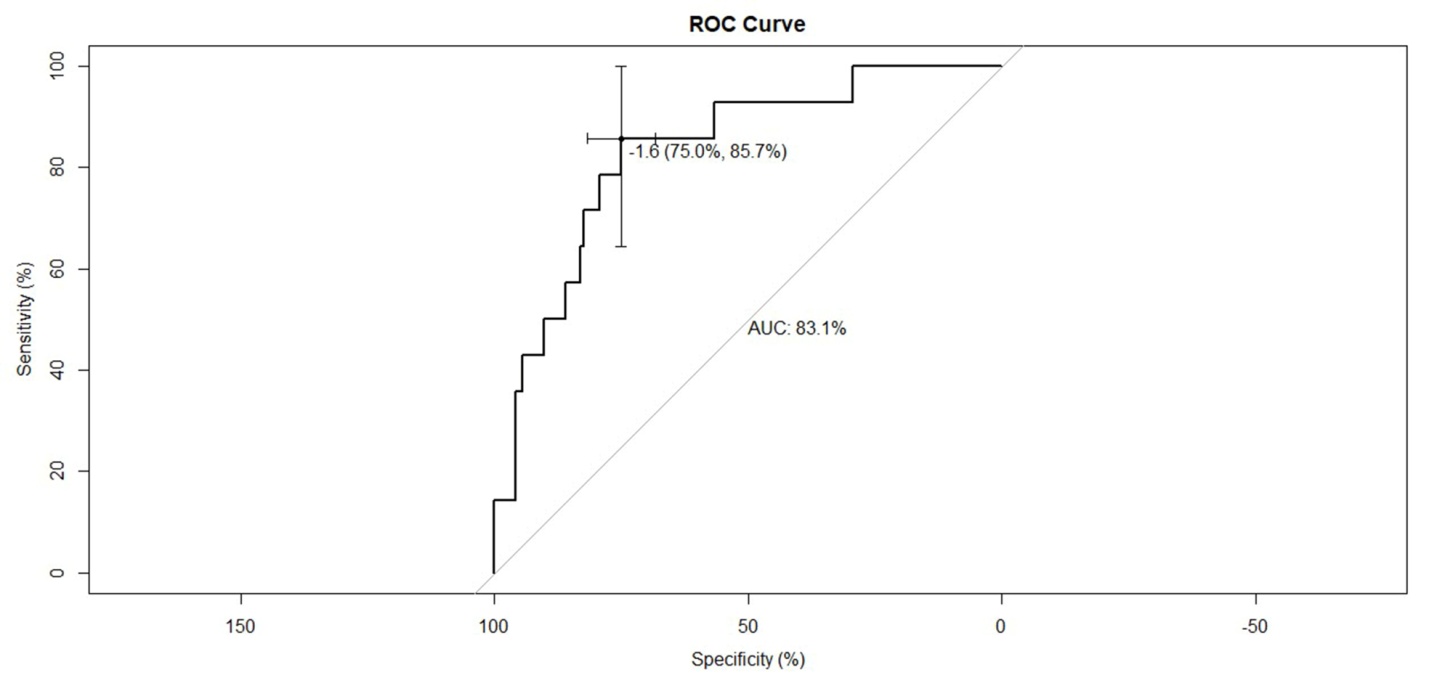
**

**Supplementary Fig 1. The discriminative performance of external (validation cohort) validation of the nomogram prediction model.** The AUC-ROC of the model was 0.831 (95% CI, 0.724–0.938), and the specificity and sensitivity were 75.0% and 85.7% respectively.
