# Supplementary material for: Nomogram to predict hemorrhagic transformation for acute ischemic stroke in Western China: a retrospective analysis
Source: BMC Neurol. 2022 Apr 26;22:156. doi: 10.1186/s12883-022-02678-2 (PMC9040382; doi:10.1186/s12883-022-02678-2)
Supplement: Supplementary file 2 — Additional file 2. [file 12883_2022_2678_MOESM2_ESM.docx]

| Groups | HT, n (%) | | Total |
| --- | --- | --- | --- |
|  | Yes | No |  |
| development cohort | 48(12.2) | 344(87.8) | 392 |
| validation cohort | 14(7.9) | 164(92.1) | 178 |
| Total | 62(10.9) | 508(89.1) | 570 |

**Supplementary Table 1. The incidence of HT in the development and validation cohorts**
