# Supplementary material for: Nomogram to predict hemorrhagic transformation for acute ischemic stroke in Western China: a retrospective analysis
Source: BMC Neurol. 2022 Apr 26;22:156. doi: 10.1186/s12883-022-02678-2 (PMC9040382; doi:10.1186/s12883-022-02678-2)
Supplement: Supplementary file 3 — Additional file 3. [file 12883_2022_2678_MOESM3_ESM.docx]

|  | Regression  coefficient | Standard  Error | Odds Ratio | 95%  Confidence Interval | P value |
| --- | --- | --- | --- | --- | --- |
| Diabetes mellitus | 0.906 | 0.409 | 2.475 | （1.109-5.521） | 0.027 |
| Atrial fibrillation | 1.737 | 0.423 | 5.678 | （2.481-12.996） | 0.000 |
| TC | -0.371 | 0.174 | 0.690 | （0.490-0.970） | 0.033 |
| Fib | 0.419 | 0.168 | 1.521 | （1.095-2.113） | 0.012 |
| Cerebral infarction Area | 0.173 | 0.052 | 1.189 | （1.073-1.317） | 0.001 |
| Cerebral infarction Volume | -0.033 | 0.015 | 0.968 | （0.940-0.996） | 0.026 |
| NIHSS score | 0.106 | 0.035 | 1.112 | （1.039-1.191） | 0.002 |
| onset-to-treatment（OTT） | 0.024 | 0.007 | 1.024 | （1.010-1.038） | 0.001 |

**Supplementary Table 2. Multivariable logistic regression analysis of the variables associated with HT in the development cohort**
