# Supplementary material for: Nomogram to predict hemorrhagic transformation for acute ischemic stroke in Western China: a retrospective analysis
Source: BMC Neurol. 2022 Apr 26;22:156. doi: 10.1186/s12883-022-02678-2 (PMC9040382; doi:10.1186/s12883-022-02678-2)
Supplement: Supplementary file 4 — Additional file 4. [file 12883_2022_2678_MOESM4_ESM.docx]

|  | Tolerance | VIF |
| --- | --- | --- |
| Diabetes mellitus | 0.980 | 1.021 |
| Atrial fibrillation | 0.818 | 1.222 |
| TC | 0.971 | 1.030 |
| Fib | 0.975 | 1.026 |
| Cerebral infarction Area | 0.089 | 11.283 |
| Cerebral infarction Volume | 0.090 | 11.071 |
| NIHSS score | 0.638 | 1.567 |
| onset-to-treatment（OTT） | 0.924 | 1.082 |

**Supplementary Table 3. Collinearity of combinations of variables in the development cohort**
