# Supplementary material for: Nomogram to predict hemorrhagic transformation for acute ischemic stroke in Western China: a retrospective analysis
Source: BMC Neurol. 2022 Apr 26;22:156. doi: 10.1186/s12883-022-02678-2 (PMC9040382; doi:10.1186/s12883-022-02678-2)
Supplement: Supplementary file 5 — Additional file 5. [file 12883_2022_2678_MOESM5_ESM.docx]

|  | Proportion of variance of coefficient | | | | | | | |
| --- | --- | --- | --- | --- | --- | --- | --- | --- |
| Condition  Index | Diabetes mellitus | Atrial fibrillation | TC | Fib | CIA | CIV | NIHSS | OTT |
| 1.000 | 0.010 | 0.010 | 0.000 | 0.000 | 0.000 | 0.000 | 0.010 | 0.010 |
| 2.033 | 0.050 | 0.040 | 0.000 | 0.000 | 0.010 | 0.010 | 0.010 | 0.060 |
| 2.738 | 0.100 | 0.600 | 0.000 | 0.000 | 0.010 | 0.010 | 0.000 | 0.000 |
| 2.947 | 0.650 | 0.060 | 0.000 | 0.000 | 0.000 | 0.000 | 0.000 | 0.210 |
| 3.882 | 0.190 | 0.200 | 0.010 | 0.020 | 0.000 | 0.010 | 0.090 | 0.520 |
| 5.132 | 0.000 | 0.030 | 0.020 | 0.020 | 0.010 | 0.010 | 0.880 | 0.190 |
| 9.190 | 0.000 | 0.000 | 0.26 | 0.770 | 0.000 | 0.000 | 0.000 | 0.000 |
| 12.953 | 0.000 | 0.060 | 0.050 | 0.010 | 0.890 | 0.870 | 0.000 | 0.000 |
| 16.560 | 0.010 | 0.010 | 0.660 | 0.180 | 0.080 | 0.090 | 0.010 | 0.010 |

**Supplementary Table 4. Collinearity of combinations of variables in the development cohort**
